# Silencing of STAT3 via Peptidomimetic LNP-Mediated Systemic Delivery of RNAi Downregulates PD-L1 and Inhibits Melanoma Growth

**DOI:** 10.3390/biom10020285

**Published:** 2020-02-12

**Authors:** Ehexige Ehexige, Mingming Bao, Purevbat Bazarjav, Xiang Yu, Hai Xiao, Shuqin Han, Huricha Baigude

**Affiliations:** Institute of Mongolian Medicinal Chemistry, School of Chemistry & Chemical Engineering, Inner Mongolia University, Hohhot 010020, Inner Mongolia, China; ehexige@mail.imu.edu.cn (E.E.); baomingming@mail.imu.edu.cn (M.B.); purevbatbazarjav@yahoo.com (P.B.); xiangyu@mail.imu.edu.cn (X.Y.); haixiao@mail.imu.edu.cn (H.X.)

**Keywords:** peptidomimetic lipid nanoparticle, RNAi, STAT3, PD-L1, cancer immune checkpoints

## Abstract

Cutaneous melanoma is the most aggressive skin cancer with notorious drug resistance. Inhibition of immune checkpoint molecules is one of the most promising approaches for cancer therapy. Herein, we show that RNAi mediated silencing of STAT3 expression in the tumor tissue robustly inhibit tumor growth in B16F10 mouse model of melanoma. We designed a peptidomimetic-based lipid nanoparticles (LNPs) for the delivery of siRNA in mouse model of melanoma. When systemically administered, the novel formulation (denote DoCh) preferentially delivered siRNA to the tumor tissue. Remarkably, sequential intravenous injections of siRNA against STAT3 induced profound silencing of STAT3 expression in tumor tissue, which resulted in significant downregulation of PD-L1, leading to significant inhibition of tumor growth through inhibition of tumor immune checkpoint. Moreover, DoCh-mediated siRNA delivery did not show noticeable damage to the major organs. Collectively, our data demonstrated that DoCh LNP is a promising tumor-targeted siRNA delivery system.

## 1. Introduction

Cutaneous melanoma is the most aggressive skin cancer which has high plasticity and drug resistance. About 75% of lethal skin cancers are malignant melanoma [1,2], the incidence of which is increasing, represent a major public health problem [3]. Chemotherapy is often ineffective for advanced malignant melanoma because of the intrinsic or extrinsic drug resistance of the cancer cells, which results, in part, from an altered apoptotic pathway following overexpression of Bcl-2 that reduces the response to anticancer agents [4]. BRAF is activated by somatic point mutation in human cancer (mutation in 66% of malignant melanoma) [5,6]. Targeted BRAF inhibition therapy (such as vemurafenib and dabrafenib) improved overall survival of melanoma patients with limited durable responses and high relapse rate (50%) [7]. Improved survival was also achieved by antibodies targeting T-lymphocyte-associated antigen 4 (CTLA-4), programmed death 1 (PD-1), and programmed death ligand-1 (PD-L1). However, intrinsically resistant tumors (such as tumors with fewer mutation loads) do not respond to immunotherapy [8]. Therefore, there is an urgent need for development of novel therapeutics for the treatment of melanoma.

RNA therapeutics target and degrade mRNA, downregulating the expression of disease related genes at posttranscriptional level. Antisense oligonucleotides (ASOs) and short interfering RNA (siRNA) have been explored for their potential for melanoma medication. SiRNA can be delivery into skin using carriers such cationic polymers or peptide enhancers [9]. Transdermal (topical) delivery of siRNA targeting BRAF to mice melanoma model using carbon nanotube functionalized with polyethylenimine (PEI) attenuated tumor growth over 25-day period [10]. siBRAF has also been delivered to basal epidermis using edge-activated liposomes for melanoma therapy [11]. Another promising target for melanoma therapy is signal transducer and activator of transcription 3 (STAT3), which has an important role in tumor cell proliferation, survival, invasion and immunosuppression [12]. SiRNA against STAT3 has been delivered to melanoma cells through lipid conjugated PEI [13], via dissolving microneedles [14] and gold nanoparticles [15], resulting in decreased STAT3 activity leading to the enhanced cancer cell apoptosis, decreased VEGF level and inhibition of tumor growth. However, efficiency of transdermal delivery of siRNA and toxicity of cationic polymers such as PEI used in the device or the formulation should be carefully evaluated.

We have created siRNA delivery systems based on nontoxic biocompatible materials including natural polysaccharides [16,17] and peptide derivatives [18,19]. Lipid functionalized peptidomimetic (DoGo3 and Chorn3) LNP highly efficiently delivered siRNA to various cells in vitro as well as in vivo without showing apparent cytotoxicity. In this report, we prepared a novel LNP formulation via optimizing the combination of both DoGo lipid and Chorn lipid (denote DoCh). We demonstrate that DoCh exhibited in vitro and in vivo siRNA delivery efficiency exceeding any of the single formulations alone. We show, for the first time in our knowledge, that systemic administration of siRNA against STAT3 complexed to DoCh LNP to B16F10 mouse melanoma model resulted in accumulation of the siRNA in tumor tissue, remarkably downregulated the expression STAT3 and the downstream gene PD-L1, and inhibited tumor growth possibly through partial elimination of immune checkpoint. Our data suggests that DoCh LNP is a promising siRNA carrier system for tumor-targeted RNAi therapy.

## 2. Materials and Methods

### 2.1. Synthesis of Lipid Functionalized Peptidomimetics

DoGo4 was synthesized according to the previous published method [19]. Briefly, DoGo1 was reacted with Boc-Orn(Boc)-OH using EDCI as condenser to give DoGo2. Then, DoGo2 was deprotected by stirring in a mixture of trifluoroacetic acid and dichloromethane. The deprotected DoGo2 was again reacted with Boc-Orn(Boc)-OH using EDCI as coupling reagent to give DoGo4, which was deprotected in trifluoroacetic acid before used in the formulation. Chorn3 was synthesized according to the previous report [18]. Briefly, ethylenediamine and cholesteryl chloroformate were reacted to give intermediate product, which was subsequently reacted with Boc-Orn[(Boc-Orn(Boc-Orn(Boc)))]-OH (GL Biochem Ltd., Shanghai, China) to give Chorn3. The deprotected Chorn3 was used in the formulation.

### 2.2. Formulation of DoCh Lipid Nanoparticle

Dogo4 was mixed with Chorn3 and mPEG DSPE (Aladdin, Shanghai, China) at molar ratio of 2:1:0.1, 3:1:0.1, 4:1:0.1 in a solution of DCM and methanol (6:1), respectively. Then, the solvent was removed under reduced pressure and the residue solid was dried under vacuum. PBS (1 X, pH = 7.4) was added to the dry residue so that the final concentration of each formulation reached 4.0 mg/mL. Then, the suspension was incubated at r.t. for 1 h, followed by a brief sonication and filtration (0.22 μm syringe filter) to give DoCh formulations. The formulated DoCh lipids were kept at 4 °C for further tests.

### 2.3. Electrophoresis Mobility Shift Assay

The DoCh/siRNA complexes formed at various nitrogen to phosphate (N/P) molar ratios were confirmed by gel mobility shift assay. Phosphate (P) represents phosphate group on RNA backbone, and nitrogen (N) represents the free amino groups on the DoCh lipids. The N/P ratio was calculated by taking into account the molar concentration of free amino groups of DoCh and the phosphate content per siRNA (Table 1). Custom synthesized siRNA powder (Takara Biotechnology Co., Ltd. Dalian, Liaoning, China) was dissolved in RNase free water at a final concentration of 50 μM. 0.5 μL of stock solution of siRNA (50 μM) was added to 4.5 μL RNase free water to make a 5.0 μL solution. To this, a volume of DoCh formulation was added. The volume (X) of each DoCh formulation used to complex with siRNA at N/P 1:1 was calculated according to the formula:

[Conc. of stock DoCh formulation (0.4 μg/μL) * X * Dogo4 content in the formulation (%)]/molecular weight of Dogo4 (988.0 g/mol)/number of free amines in Dogo4 (4)] + [Conc. of stock DoCh formulation (0.4 μg/μL) * X * Chorn3 content in the formulation (%)]/molecular weight of Chorn3 (961.03 g/mol)/number of free amines in Chorn3 (4)] = micromole of phosphate in siRNA (0.5 μM * 0.5 μL * 10^−6^ *40 = 0.001 μM).

N/P ratios 2, 3, 4, 5, 6 were prepared by changing the volume proportion of DoCh. The mixtures were incubated at r.t. for 20 min. Then, siRNA loading buffer was added, and the samples were run on a 1.0% agarose gel for 15 min at 100 V.

### 2.4. Measurements of Particle size of DoCh Nanoparticles

DoCh LNP (4mg/mL, 20 μL in 1 X PBS, pH = 7.4) was complexed with 5 μL siRNA (50 μM) and the complexes were incubated for 20 min at room temperature. Then, 1 mL 1X PBS, (pH = 7.4) was added to the complex, and the particle size and zeta potential were measured on a Zetasizer Nano S instrument (Malvern Instrument, UK). Final concentration of the DoCh was 80 μg/μL and siRNA was 250 nM, respectively. Dip cell was used for the measurement of zeta potential, measurement angle was 90° and measurement position (mm) was 4.50. For all the samples, attenuator was ranged from 6 to 11, dispersant RI was 1.330, viscosity (cP) was 0.882, dispersant Dielectric Constant was 78.5 and temperature was 25.0 °C.

### 2.5. Transmission Electron Microscopy (TEM)

Shape and surface morphological examination of siRNA/DoCh LNP was observed by transmission electron microscopy (Field Electron and Ion Company, Hillsboro, OR, USA). A mixture of DoCh and siRNA was incubated at r.t. for 20 min. Then, two drops of sample were placed on a copper grid and air-dried for 10 min, followed by negative staining with 2% phosphomolybdic acid solution for 2 min. The grid was allowed to air-dry for 10 min and the morphology of the LNP was examined under the TEM (Instrument: FEI Tecnai G2 F20. Voltage: 200kV).

### 2.6. Cell Culture and Cytotoxicity Assay

B16F10 cells, HepG2 cells, 293T cells, HeLa cells (ATCC, Manassas, VA, USA) were maintained at 37 °C with 5% CO_2_ in medium (BIOIND), supplemented with 10% fetal bovine serum, 100 U/mL penicillin and 100 μg/mL streptomycin (Gibco). B16F10-GFP cells (AntiCancer, Inc., San Diego, CA, USA) were maintained in the 400 μg/mL concentration of G418 (GENETICIN; Gibco). For cytotoxicity assay, cells were seeded in 96-well plates at 5000 cells/well in 100 μL of culture medium. 24 h after seeding, DoCh LNP (4 mg/mL) was added to the cells at final concentrations of 4, 8, 20, 60 and 100 μg/mL in triplicate. After 24 h, cell viability was assessed using MTT assay.

### 2.7. In Vitro Transfection

For cellular uptake assay, fluorescence signal was measured by flow cytometry assay. 3.0 × 10^6^ B16F10 cells were treated with FITC-siRNA/DoCh in a 12-well plate for 6 h. Lipofectamine 2000 (L2K) was used as the comparison group. The final concentration of DoCh was 32 μg/mL. Cells were treated with FITC-siRNA/DoCh for 6 h and then fixed with 4% paraformaldehyde, followed by staining with DAPI for 10 min at the r.t. Images of cellular uptake were captured by confocal microscope (Carl Zeiss LSM710). For plasmid transfection assay, 1μg pcDNA3-eGFP was diluted in 100 μL Opti-MEM. This solution was mixed with 100 μL medium containing DoCh lipids, and incubated for 20 min before adding to the cells in 800 μL complete medium in a well of 12-well plate. The final concentration of DoCh in the well was 32 μg/mL. After 48 h, the GFP expression was evaluated by observation under fluorescence microscope. For flow cytometric analysis (FCM), cells were trypsinized, washed and resuspended in PBS and assessed on a NovoCyte Benchtop Flow Cytometer (ACEA Biosciences, Inc., San Diego, CA, USA). For transfection of siRNA, siGenome Non-targeting siRNA Control were purchased from Dharmacon (Lafayette, CO, USA). STAT3 siRNA was custom synthesized by TAKARA Biotechnology Co., Ltd. (Dalian, Liaoning, China). The sequence of STAT3 siRNA: sense strand: 5′-GGACGACUUUGAUUUCAACTT-3′; antisense strand: 5′-GUUGAAAUCAAAGUCGUCCTG-3′. Cells were seeded in 12-well plate at 3.0 X 10^6^ cell/well density and cultured for 24 h. When cell confluency reached 65%, cells were transfected with either DoCh/siSTAT3 siRNA complex, or DoCh/siGenome Non-targeting siRNA Control at siRNA concentration of 5 nM, 10 nM, 25 nM, respectively. The final concentration of DoCh in the well was 32 μg/mL. After 24 h, total RNA was extracted using TRIZOL (Invitrogen, Carlsbad, CA, USA) and the expression of STAT3 mRNA was measured with iScriptTM Reverse Transcription Supermix for RT-qPCR and iTaqTM Universal SYBR Green Supermix (Bio-Rad, Hercules, CA, USA) for quantitative PCR. The sequence of primers used for RT-qPCR were as following: mouse STAT3, forward: 5′-GTTCCTGGCACCTTGGATT-3′, reverse: 5′-CAACGTGGCATGTGACTCTT-3′. Mouse PD-L1, forward: 5′-GTTGTTCCTCATTGTAGTGTCCA-3′, reserve: 5′-CACATTTCTCCACATCTAGCATTC-3′. Mouse β-actin, forward: 5′-CATTGGCCAACCGTGAAAAG-3′, reverse: 5′-ACCAGAGGCATACAGGGACA -3′.

### 2.8. Western Blotting

Total protein was extracted using M-PER™ (Thermo scientific, Waltham, MA, USA) for further test. Cell lysate or tissue homogenate were diluted to 2 mg/mL. SDS-PAGE gel was prepared with Invitrogen SureCast™ solution. 20 μg of protein was loaded into the wells of SDS-PAGE gel, and ran the gel at 80 V for 20 min, then at 120 V for 2 h. Then the protein was transferred to activated PVDF membrane (Immobilon-P Transfer Membrane, 0.45 μm) with Trans-Blot Turbo Transfer system (Bio-Rad, Hercules, CA, USA), at 25 V, 2.5 A for 20 min. Then, the membrane was blocked for 4 h using 5% blocking solution (5% milk/TBST buffer). After blocking, primary and secondary antibody staining was performed using recommended dilutions in 5% blocking solution at r.t. for 2 h. After antibody staining, membranes were washed 3 times using 2% milk/TBST for 10 min. Images of Western blotting were captured with SuperSignal™ West Pico (Thermo Scientific^TM^) using UVP Chemstudio (Analytic Jena). STAT3 antibody (79D7) (1:2000) Rabbit mAb #4904; GAPDH antibody (14C10) (1:1000) Rabbit mAb #2118 were from Cell Signaling Technology (Danvers, MA, USA). PD-L1 antibody (1:1000) Rabbit Anti-PD-L1 antibody #ab213480; secondary antibody (1:4000) Goat Anti-Rabbit IgG H&L (HRP) #ab97051 were from Abcam.

### 2.9. Animals and Tumor Model

C57BL/6 mice were obtained from Vital River Laboratories (Beijing, China) and were housed in plastic cages under controlled conditions and maintained according to the Guide for the Care Use of Laboratory Animals established by Inner Mongolia University (IMU, Hohhot, China). The project identification code is: 21875124, date of approval is: August 23, 2019, and name of the ethics committee is: The Institutional Animal Care and Use Committee at Inner Mongolia University. To make tumor-bearing mouse models, 1.0 × 10^6^ B16F10-GFP cells 50 μL in culture medium were injected subcutaneously into the left back flank region. Cell suspension became subcutaneous solid tumor at the 8th day after injection. To monitor the tumor growth by live imaging and to collect tissues at the end of the experiment period, mice were divided into 4 groups (*n* = 8 mice per group) according to tumor volume and body weight randomly. To the siSTAT3/DoCh treatment group, 2 mg/kg body weight of siRNA and 20 mg/kg body weight of DoCh lipid nanoparticles were complexed at r.t. and injected via the tail vein from day 8 once a week. Adriamycin (ADR) is an anthracycline class of anti-cancer drug and have a broad spectrum of activity in human tumors as well as animal model of tumor. Therefore, we used ADR as a positive control for the in vivo antitumor experiments. To the ADR (Aladdin, Shanghai, China) group, ADR (2 mg/kg) was intraperitoneally injected twice a week. To the siControl/DoCh group, siGenome Non-targeting siRNA Control and DoCh lipid nanoparticles complex were injected in the same way as siSTAT3/DoCh group. Nontreated group was negative control group. Tumor size and body weight were measured every 2 days from day 8. The mice were anesthetized with isoflurane by a respiratory route and whole body live image of mice were obtained with UVP Ibox Scientia^TM^ (Analytik Jena, Jena, Germany) at day 8, 15, 22. At day 22, mice were anesthetized, then tumors, liver, lung, and serum were collected and immediately frozen in liquid nitrogen and stored at −80 °C for further study. For survival rate experiment (*n* = 12 mice per group), treatments were same as tumor collecting experiment and survival rates of the mice were observed and calculated for 60 days. For qPCR assay, siControl/DoCh, siSTAT3/DoCh treated mice were (*n* = 3 mice per group) compared with nontreated mice in triplicate and tumors collected at day 9 and 16. For Western blotting assay, tumors were collected at day 16.

### 2.10. Biodistribution Assay

For biodistribution study, B16F10 cells were injected to make tumor-bearing mouse model. Other detail was in the same way of B16F10-GFP tumor model. 2 mg/kg FITC-siRNA and 20 mg/kg DoCh lipid nanoparticles were complexed at r.t. and injected via the tail vein at day 12. After 24 h, mice were sacrificed, and the organs and tumors were harvested. Naked FITC-siRNA was i.v. injected as control. Ex in vivo fluorescence image of the tumor and major organs were obtained using UVP Ibox Scientia^TM^ (analytic jena). 0.5 g of tumor and organs were homogenized with liquid nitrogen and then the fluorescence signal was obtained using UVP Ibox Scientia^TM^. Fluorescence signal of gradient concentration of FITC-siRNA were obtained with UVP Ibox Scientia^TM^, then fluorescence intensity was given by Image J through pixel values to establish a standard curve line to quantify each organs’ fluorescence signal. Tumor was cut into 5 μm using Leica CM 1950 then fixed with 4% paraformaldehyde onto the slide. Tissue slide was stained using DAPI and Phalloidin-iFluor 555 sequentially. Images were obtained using confocal microscope (Carl Zeiss LSM710) at the channel of Ex/m = 556/574 nm; 460 nm/550 nm; Ex/m = 358/461 nm.

### 2.11. Blood Chemistry and HE Staining

The level of liver enzymes (alanine aminotransferase (ALT), aspartate aminotransferase (AST)) in the serum of mice treated with DoCh LNP were measured using Alanine Aminotransferase Kit and Aspartate Aminotransferase kit, respectively (Meikang BioScience, Ningbo, China). For histochemical analysis of liver and lung, method of Hematoxylin-Eosin (H&E) staining and tissue slide making were mentioned above. The images were obtained with EVOS XL Core microscope (Invitrogen, Carlsbad, CA, USA).

### 2.12. STAT3 and PD-L1 Knockdown In Vivo

For in vivo siRNA delivery by DoCh LNP, siSTAT3/DoCh LNP or siControl/DoCh were administrated (*n* = 6 in each group) to B16F10-GFP tumor-bearing mouse model via i.v. injection at day 8. After 24 h, tumor tissue (*n* = 3 of each group) were collected. At day 15, siSTAT3/DoCh LNP or siControl/DoCh was i.v. injected into the remaining 3 mice in each group. After 24 h, tumor tissues were collected. All samples were immediately frozen in liquid nitrogen and stored at −80 °C for further study. Method of qPCR and Western blotting were mentioned above.

### 2.13. Statistical Analysis

The data were expressed as the means ± SEM and were analyzed for significant differences by one-way analysis of variance (ANOVA) with a Dunnett’s multiple comparisons test using GraphPad Prism 7.0. Differences were considered statistically significant if *p* value is <0.05.

## 3. Results and Discussion

**Figure 1:**
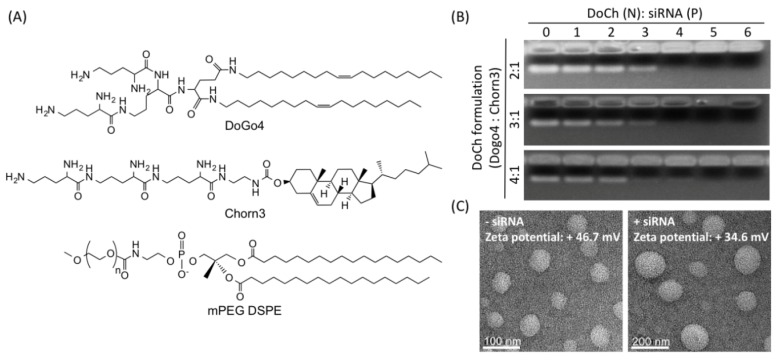
Formulation and characterization of DoCh LNP. (**A**) Composition of DoCh LNP formulation; (**B**) Agarose gel image showing siRNA binding affinity of DoCh LNP formulations; (**C**) TEM images of DoCh LNP before and after binding with siRNA.

In our previous studies, we discovered that configuration of peptidomimetic headgroup of cationic lipids can improve the nucleic acid delivery efficiency and decrease cytotoxicity [20]. To explore the potential of tumor-targeted siRNA delivery of the peptidomimetic LNP, we designed novel formulations which mainly contain DoGo4 [19] and Chorn3 [18] at three different ratios, i.e., 2:1, 3:1 and 4:1 (Figure 1A). The formulation (denote DoCh) is predominantly hydrophilic, nonetheless, all three formulations formed LNP with diameters ranging from 50 nm to 80 nm (Figure 1B). Moreover, all three formulations efficiently encapsulated siRNA to give LNP with diameter ranging from 100 nm to 180 nm (Figure 1B, bottom). The zeta potential of the LNP (DoGo4:Chorn3, 4:1) which was +46.7 mV decreased to +34.6 mV upon loading of siRNA (Table 2). DoCh LNPs contain nearly pure cationic lipids compared to the traditional liposome which normally contain cationic lipid, cholesterol and neutral lipid. However, inclusion of mPEG DSPE at 1%mol stabilized the LNP and decreased the zeta potential by the shielding charge [21].

**Figure 2:**
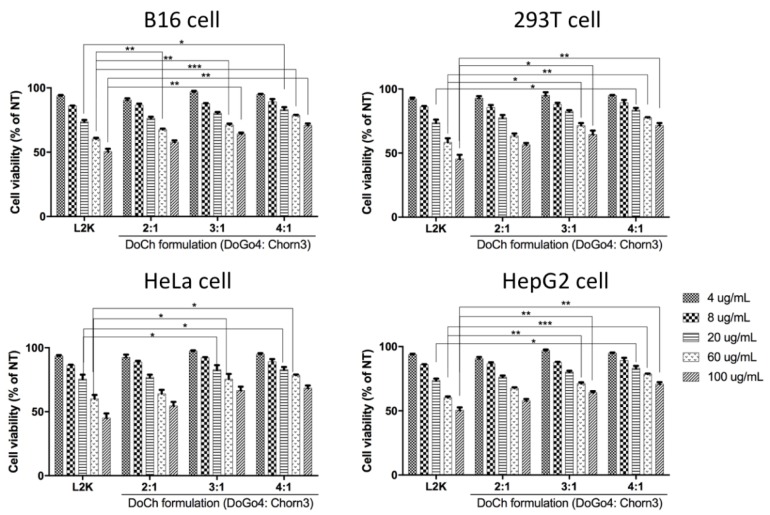
Measurement of cytotoxicity of DoCh formulations. Cells were treated with L2K or DoCh formulations at indicated concentrations for 24 h. Cell viability was measured by MTT assay. *p value is < 0.05; **p value is < 0.01, ***p value is < 0.001.

Increased percentage of DoGo4 in DoCh LNP not only enhanced siRNA binding efficiency, but also remarkably decreased cytotoxicity of the resulting LNP formulations. DoCh LNP-treated (concentrations ranging from 4 μg/mL to 100 μg/mL) B16F10 cells showed significantly higher viability compared to the cells treated with lipofectamine 2000 (L2K). At the highest concentration used for the test, cell viability was only about 50% in L2K-treated B16F10 cells, while about 75% cell viability was observed in cells treated with DoCh LNP (4:1). Amongst DoCh LNP formulations, cytotoxicity gradually decreased with the increase of DoGo4/Chorn3 ratio (Figure 2). Similar trends in cell viability were observed in all four cell lines treated with the control (L2K) and DoCh LNP formulations, indicating that DoCh LNP containing 80% DoGo4 cationic lipid has the lowest cytotoxicity.

**Figure 3:**
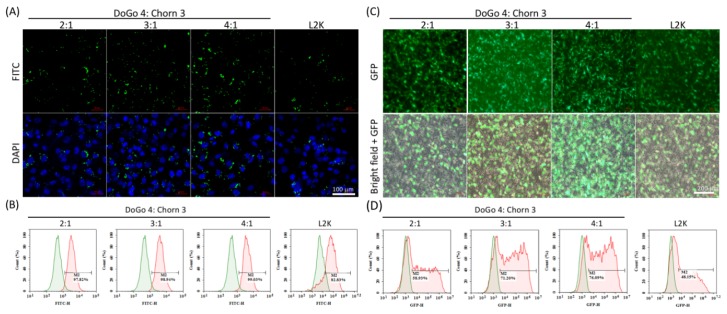
Cellular uptake of nucleic acid complexed to DoCh LNP composed of different ratio of DoGo4 and Chorn3. (**A**) Confocal images of B16F10 cells treated with FITC-siRNA/DoCh LNP complexes; (**B**) Quantification of B16F10 cellular uptake of FITC-siRNA/DoCh LNP complexes by flow cytometry; (**C**) Fluorescence images of B16F10 cells treated with pcDNA-eGFP/DoCh LNP complexes; (**D**) Quantification of GFP expression of B16F10 cells treated with pcDNA-eGFP/DoCh LNP complexes by flow cytometry.

To assess the in vitro nucleic acid delivery efficiency of DoCh LNPs, we first treated B16F10 cells with FITC-labeled siRNA complexed to DoCh LNPs, respectively. After 24 h, the FITC signal of the treated cells was observed under fluorescence microscope, and subsequently, the FITC-positive cell numbers were quantified using flow cytometry. Over 90% B16F10 cells were counted as FITC-positive cells for all three DoCh LNPs, slightly higher than L2K (Figure 3A,B). However, when B16F10 cells were transfected with an GFP-expressing plasmid complexed to DoCh LNPs, DoCh (3:1, 71%) and DoCh (4:1, 76%), it showed significantly higher GFP-positive cells compared to DoCh (2:1, 58%) as well as L2K (48%) (Figure 3C,D), suggesting that DoCh LNPs with higher DoGo4 content may have better cell penetration ability as well as cargo-releasing efficiency in the cytoplasm.

**Figure 4:**
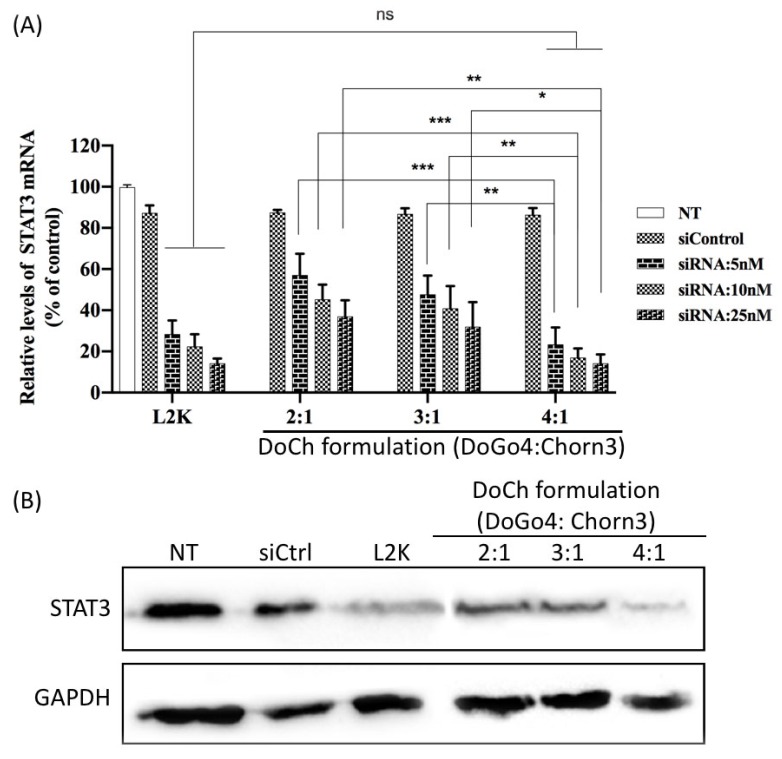
Quantification of in vitro siRNA delivery efficiency of DoCh formulations. (**A**) Measurement of STAT3 mRNA level in B16F10 cells after transfection with control siRNA (siCtrl) or siRNA against STAT3 complexed to either lipofectamine (L2K) or DoCh with different formulations. (**B**) Measurement of STAT3 protein level by Western blot in B16F10 cells after transfection with control siRNA (siCtrl) or siRNA against STAT3 complexed to either lipofectamine (L2K) or DoCh with different formulations. siRNA concentration: 25 nM. *p value is < 0.05; **p value is < 0.01; ***p value is < 0.001.

To further evaluate siRNA delivery efficiency of DoCh LNPs, we transfected B16F10 cells with increasing concentration of siRNA against STAT3 complexed to DoCh LNPs, respectively. After 24 h, we extracted total RNA from the cells, and mRNA level as well as protein level of STAT3 was quantified by RT-qPCR and Western blot, respectively. As shown in Figure 4, DoCh (4:1) showed the best RNAi, equivalent to L2K, reaching 90% knockdown of STAT3 at siRNA concentration of 25 nM.

**Figure 5:**
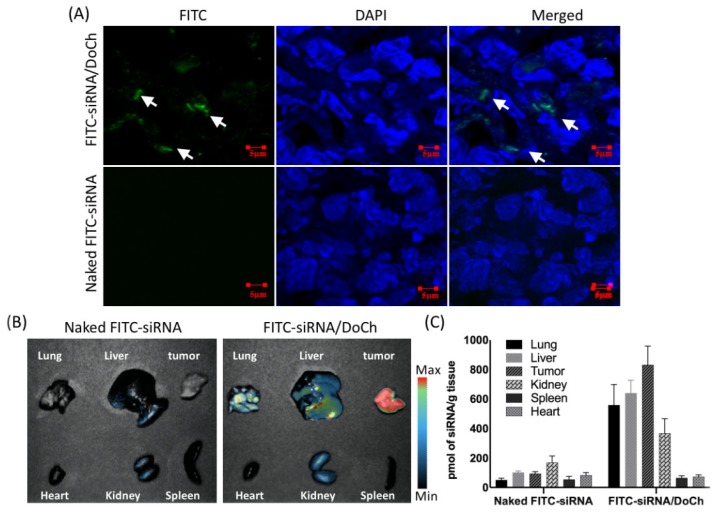
Distribution of siRNA in B16F10 tumor tissue. (**A**) Naked FITC-siRNA or FITC-siRNA complexed to DoCh LNP was i.v. injected to B16F10 mouse melanoma model, and the siRNA signal was detected in tumor tissue slide by fluorescence microscope. White arrows indicate FITC signal. (**B**) Organ distribution of FITC-labeled siRNA complexed to DoCh LNP (left) and naked FITC-labeled siRNA (right). (**C**) Quantification of siRNA organ distribution in B16F10 melanoma model.

Next, we evaluated tumor-targeting efficiency of DoCh LNP in B16F10 mouse melanoma model. To do this, we first administered FITC-labeled siRNA alone, or FITC-siRNA/DoCh LNP to B16F10 tumor model by i.v. injection. After 24 h, we dissected the tumor tissue, and observed FITC signal distribution within the tumor tissue by immunohistochemistry (IHC) analysis. While there was no fluorescence signal detected in tumor from the mouse administered with naked FITC-siRNA, apparent fluorescence particles were detected in the mouse injected with FITC-siRNA complexed to DoCh LNP (Figure 5A), confirming that DoCh LNP not only deliver siRNA to tumor tissue but also penetrate the tumor tissue and release siRNA cargo in the cytoplasm of tumor cells. To assess the absorption of the DoCh LNP by organs other than tumor tissue, we dissected liver, lung, spleen, heart and kidney, as well as tumor from the mouse administered with FITC-siRNA/DoCh LNP complex, and observed and measured the fluorescence signal from the tissues using UVP Ibox Scientia^TM^. Only trace amount of fluorescence signal was detected in liver and kidney of mouse injected with naked siRNA. The strongest signal was detected in the tumor of mouse administered with FITC-siRNA/DoCh LNP, followed by liver, lung and kidney (Figure 5B,C), indicating the preferable uptake of siRNA/DoCh LNP by tumor.

**Figure 6:**
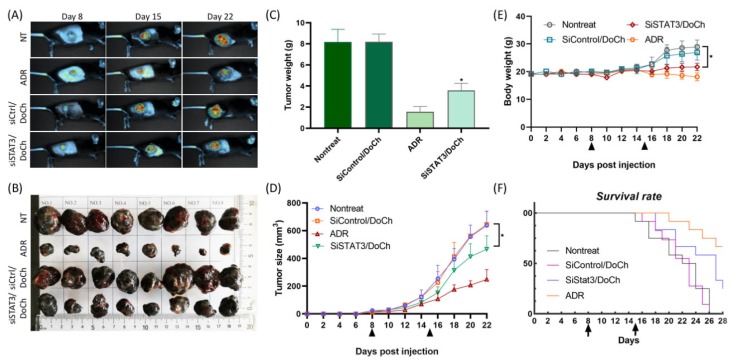
Antitumor efficiency of DoCh LNP mediated tumor-targeted siRNA delivery. (**A**) Live animal imaging and (**B**) images of dissected tumor of nontreated (NT), ADR treated, siSTAT3/DoCh and siCtrl/DoCh treated B16F10 mouse melanoma model. Live animal images were taken at day 8, day 15 and day 22, and tumor images were taken from dissected tumors at the end of experiment (day 22). Body weight (**C**), tumor volume (**D**) and tumor weight (**E**) of mice treated with siSTAT3/DoCh LNP decreased, while survival rate significantly extended (**F**). ADR, Adriamycin. *p value is < 0.05.

Next, we observed the tumor growth in nontreated (NT) mice and mice treated with DoCh LNP, either complexed to a control siRNA (siCtrl) or siSTAT3, as well as a positive control adriamycin (ADR). To do this, we first established B16F10 melanoma model by subcutaneously injecting B16F10 cells permanently expressing GFP. At day 8, tumor growth between four different groups was not noticeable. However, by day 15 and day 22, significantly reduced tumor was observed in B16F10 tumor treated with siSTAT3/DoCh LNP (Figure 6A,B). Efficient knockdown of STAT3 and downregulation of PD-L1 expression in tumor resulted in the decrease of tumor weight and tumor volume. The body weight of the tumor animals was decreased compared to nontreated animals, but in a lesser extent compared to the body weight of ADR-treated animals, which may have resulted from combined effect of the toxicity of the drug as well as decrease of the tumor weight (Figure 6C–E). Moreover, the survival rate of the tumor model mice treated with siSTAT3/DoCh LNP was significantly extended (Figure 6F), suggesting the therapeutic effect of DoCh-mediated knockdown of STAT3. The mechanism of action of small molecular drug and siRNA therapeutics is different. We used ADR dose according to previous reports [22] that confirmed the effectiveness of such control. Dose of DoCh LNP depends on characteristics of siRNA delivery, which is involved in a number of biological processes. Although we using ADR as a positive control, in some conditions their comparison is limited. DoCh LNP can be given 2× per week, but our research goal is to investigate siRNA delivery capability of DoCh LNP. Chemically-modified siRNA was used clinically and proved to be more stable and potent with administration frequency of 1× per 3 weeks. Referring to this, we decided to test the efficiency of once a week i.v. injection of siSTAT3/DoCh LNP.

**Figure 7:**
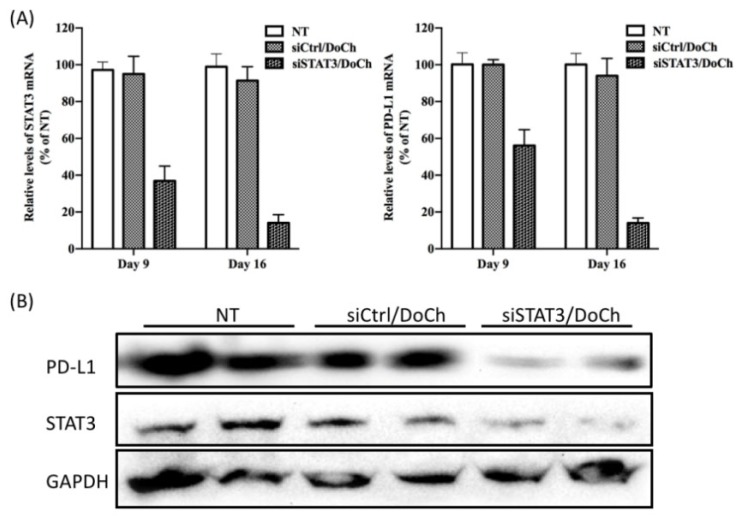
Measurement of the efficiency of DoCh mediated tumor-targeted RNAi delivery. (**A**) RT-qPCR analysis of STAT3 and PD-L1 expression in the tumor tissue after i.v. administration of siSTAT3/DoCh LNP. Values represent the mean ±SEM of tissue samples from 3 tumor regions (4 animals). Data are expressed as percent of control. (**B**) STAT3 and PD-L1 protein levels are reduced in tumor of mice injected with siSTAT3/DoCh LNP. STAT3 and PD-L1 protein expression levels were measured by Western blot 16 days after the injection of 2 mg/kg body weight of siSTAT3 or its control complexed to DoCh, respectively.

To investigate siSTAT3/DoCh LNP-induced RNAi efficiency, we extracted total RNA as well as total protein from tumor dissected from each group of mice one day after the first injection (day 9) and the second injection (day 16), respectively. A subsequent analysis of gene expression level by RT-qPCR revealed that, at day 9, STAT3 mRNA decreased 65% and at day 16, 90% knockdown of STAT3 was observed (Figure 7A). Meanwhile, the expression of immune checkpoint molecule PD-L1 was also significantly decreased. Moreover, the protein level of both STAT3 and PD-L1 were significantly decreased one day after the second injection (Figure 7B), further confirming the efficiency of DoCh LNP-mediated tumor-targeted delivery of RNAi and the cellular pathway as well as phenotypic changes induced by such RNAi effects.

**Figure 8:**
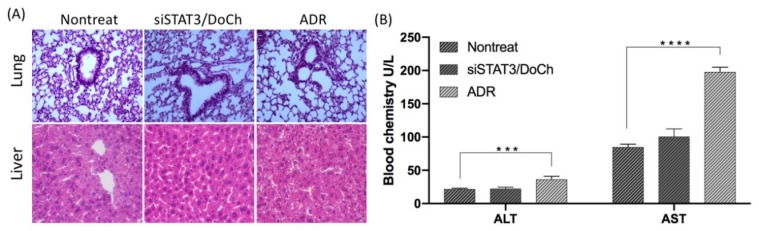
Assessment of DoCh LNP toxicity. (**A**) H&E staining of lung and liver from nontreated, siSTAT3/DoCh treated and ADR treated mice. Magnification: ×400. (**B**) Measurement of serum ALT and AST from nontreated, siSTAT3/DoCh treated and ADR treated mice. ***p value is < 0.001; ****p value is < 0.0001.

According to the biodistribution assay, we learned that siRNA/DoCh LNP were strongly accumulated in the tumor, liver and lung compared to the naked siRNA group. To evaluate the possible toxic effect of DoCh-mediated RNAi delivery, we performed histological assessment of liver and lung of the tumor-bearing mice at day 22. No visible lesions in the lung and liver in nontreated and siSTAT3/DoCh-treated group (Figure 8A), indicating that there were no significant acute toxic effects caused by DoCh LNP in vivo. Liver and lung histology of ADR treatment group shows severe mononuclear cell infiltration indicating that long-term injection of ADR may lead to significant toxicity in vivo. Moreover, measurement of serum ALT/AST level showed that nontreated and siSTAT3/DoCh-treated group had normal level of the liver enzymes, while ADR group showed elevated level of both enzymes due to the toxicity (Figure 8B). These data suggest that, while a therapeutic dose of siRNA/DoCh LNP was injected, it might eventually undergo a biocompatible degradation process in vivo.

## 4. Conclusions

In this report, we show that DoCh LNP formulated by incorporation of DoGo4, Chorn3 and mPEG DSPE had excellent siRNA transfection efficiency in vitro with negligible cytotoxicity. In B16F10 mouse melanoma model, systemic delivery of siSTAT3/DoCh LNP resulted in preferential accumulation of the siRNA in the tumor. Moreover, DoCh-mediated delivery of siSTAT3 induced 90% knockdown of STAT mRNA in the tumor tissue after two sequential injections, leading to the downregulation of immune checkpoint molecule PD-L1 in the tumor and significant inhibition of tumor growth. Collectively, our results provide a promising tumor-targeting nucleic acid delivery system.

## Figures and Tables

**Table 1:** Calculation of volume (μL) of each DoCh stock solution used to form N/P (1:1).

DoCh (Dogo4: Chorn3:PEG^(a)^)	Stock Solution (μL) ^(b)^	RNase Free H_2_O (μL)	Total Amine^-^ (N, nmol)	Stock siRNA (μL) ^(c)^	Total Phosphate^-^ (P, nmol)	RNase Free H_2_O (μL)	N/P
2:1:0.1	0.63	4.37	1.0	0.5	1.0	4.50	1.0
3:1:0.1	0.62	4.38	1.0				
4:1:0.1	0.62	4.38	1.0				

(a) mPEG DSPE. (b) Stock solution DoCh formulation (Dogo4 and Chorn3 contains 4 free amino groups respectively): 0.4 mg/mL. (c) Stock solution of siRNA (40 nucleotides double strand): 50 μM.

**Table 2:** Particle size and zeta potential of DoCh formulations before and after siRNA loading.

No.	Composition^(a)^	Particle Size (d.nm) ^(b)^		Zeta Potential (mV)	
		−siRNA (PDI)	+siRNA (PDI)	−siRNA	+siRNA
1	2:1:0.1	122.7 (0.23)	164.8 (0.22)	50.2 ± 2.4	32.9 ± 2.3
2	3:1:0.1	89.67 (0.22)	154.3 (0.27)	43.4 ± 1.7	35.2 ± 1.9
3	4:1:0.1	88.58 (0.21)	171.8 (0.25)	46.7 ± 2.1	34.6 ± 2.3

(a) Molar ratio of DoGo4: Chorn3: mPEG DSPE; (b) Measured by dynamic light scattering.
